# IL-10 Protects Mice From the Lung Infection of *Acinetobacter baumannii* and Contributes to Bacterial Clearance by Regulating STAT3-Mediated MARCO Expression in Macrophages

**DOI:** 10.3389/fimmu.2020.00270

**Published:** 2020-02-21

**Authors:** Min-Jung Kang, Ah-Ra Jang, Ji-Yeon Park, Jae-Hun Ahn, Tae-Sung Lee, Dong-Yeon Kim, Moo-Seung Lee, Seungwoo Hwang, Yu-Jin Jeong, Jong-Hwan Park

**Affiliations:** ^1^Laboratory Animal Medicine, College of Veterinary Medicine, Chonnam National University, Gwangju, South Korea; ^2^Infectious Disease Research Center, Korea Research Institute of Bioscience and Biotechnology, Daejeon, South Korea; ^3^Korean Bioinformation Center, Korea Research Institute of Bioscience and Biotechnology, Daejeon, South Korea

**Keywords:** *Acinetobacter baumannii*, interleukin-10, macrophages, macrophage receptor with collagenous structure, STAT3

## Abstract

Interleukin-10 plays important, yet contrasting, roles in host protection against bacterial infections and in the septic response. To determine the role of IL-10 in the host defense against *Acinetobacter baumannii* infection, wild-type (WT) and IL-10-deficient mice were infected intranasally with the bacteria. IL-10-deficient mice exhibited increased mortality, severe pathology, and excess production of proinflammatory cytokines and chemokines in the lungs, and increased bacterial burdens in bronchoalveolar lavage (BAL) fluids and lung homogenates after *A. baumannii* infection, compared to WT mice. Intranasal administration of recombinant IL-10 rescued mice from the lethality of the bacterial infection by promoting bacterial clearance and reducing production of cytokines and chemokines in the lungs. *In vitro* experiments revealed that IL-10 enhanced phagocytosis and bacterial killing by macrophages by upregulating the macrophage receptor with collagenous structure (MARCO). In addition, *A. baumannii*-induced activation of STAT3 was impaired in IL-10-deficient macrophages, which was essential for expression of MARCO. Intranasal adoptive transfer of WT macrophages resulted in significant increases in mice survival and bacterial clearance in IL-10-deficient mice infected with *A. baumannii*. Our results show that IL-10 played an important role in the host defense against pulmonary infection of *A. baumannii* by promoting the antibacterial function of macrophages by regulating MARCO expression through the STAT3-mediated pathway.

## Introduction

*Acinetobacter baumannii* is a ubiquitous, gram-negative, and opportunistic pathogen with antibiotic resistance that often causes high morbidity and mortality, especially in patients with weakened immune systems (1–3). *A. baumannii* infection can cause nosocomial pneumonia, bloodstream infections, septicemia, urinary tract infections, and meningitis (4). Treatment for *A. baumannii* infection has become increasingly difficult due to the emergence of multiple antibiotic resistance (5). Despite its clinical importance, relatively little is known about the innate immune mechanism involved in the host resistance to *A. baumannii* infection.

Interleukin-10 (IL-10) is an important anti-inflammatory cytokine produced under different conditions of immune activation by a variety of cell types, including T cells, B cells, and monocytes/macrophages (6). IL-10 seems to be a double-edged sword in the host defense against pulmonary bacterial infections and its role likely depends on the bacterial strain. Inhibition of IL-10 by a neutralizing antibody improved survival of mice infected with *Klebsiella pneumonia*, which was concluded result from enhanced bacterial clearance and increased production of pro-inflammatory cytokines (7). Lung-specific IL-10-overexpressing mice exhibited impaired bacterial clearance in the lungs and increased mortality when infected with *Pseudomonas aeruginosa* (8). Moreover, IL-10 also exhibited a harmful effect on *in vivo* protection against *Bordetella parapertussis* infection by limiting IFN-γ production, which is critical for T cell responses required to promote bacterial clearance (9). In contrast, IL-10 contributed to host protection from lethality in mice infected with *Corynebacterium kutscheri* or *Streptococcus pneumonia* by alleviating lung inflammation, although it did not affect the clearance of those bacteria (10, 11). IL-10 also demonstrated host benefit by maintaining homeostasis and regulating apoptotic neutrophil clearance in a mice model of *K. pneumonia* infection (12). Moreover, in an *E. coli*-induced meningitis model in mice, IL-10 promoted CR3-mediated bacterial clearance by phagocytosis by reducing prostaglandin E2 (PGE_2_) production (13). Taken together, it is likely that IL-10 is involved in host defenses against bacterial infections with various phenotypes and mechanisms, depending on the bacterial species.

A recent study showed that the receptor for advanced glycation end products (RAGE) suppressed IL-10 production in a systemic infection model of *A. baumannii*, which resulted in increased mortality of the infected mice, although the RAGE signaling did not affect lung inflammation or bacterial clearance when the mice were intranasally infected (14). Administration of recombinant IL-10 rescued wild type (WT) mice from *A. baumannii*-induced lethality (14), suggesting that IL-10 may play a beneficial role in host defenses against *A. baumannii* infection. However, there is no evidence on the role of IL-10 in host protection against lung infections with *A. baumannii*, and the underlying mechanism is still unclear. In the present study, we showed that IL-10 was critical for host protection against lung infection with *A. baumannii* by suppressing the lung pathology and by promoting phagocytosis and bacterial killing by macrophages through a macrophage receptor with collagenous structure (MARCO)-dependent pathway.

## Materials and Methods

### Mice

WT C57BL/6J mice were obtained from the Central Lab Animal (Seoul, Korea). IL-10-deficient mice on a C57BL/6J background were purchased from the Jackson Laboratory (Bar Harbor, ME, USA). Protocols for animal studies were approved by the Institutional Animal Care and Use Committee of Chonnam National University (Gwangju, Korea) (Approval number: CNU IACUC-YB-2015-33).

### Reagents and Antibodies

Recombinant murine IL-10 was obtained from PeproTech (Rocky Hill, NJ, USA). Total STAT3, phospho-STAT3, total AKT, and phospho-AKT antibodies were from Cell Signaling Technology (Beverly, MA, USA). The β-actin antibody was purchased from Santa Cruz Biotechnology (Santa Cruz, CA, USA). The neutralizing antibody to MARCO and goat IgG antibody were purchased from R&D Systems (Minneapolis, MN, USA). The D,15-DPP Stat3 inhibitor was obtained from Sigma Aldrich (St. Louis, MO).

### Bacterial Preparation

The KCCM 35453 *A. baumannii* strain (ATCC 15150) was purchased from the Korean Culture Center of Microorganisms (Seoul, Korea). Single colonies were inoculated into 10 ml of Luria-Bertani broth supplemented with ampicillin (50 μg/ml) and grown overnight at 37°C with 200 rpm shaking. A 1:5 dilution of the culture suspension was allowed to grow in fresh medium at 37°C with shaking at 200 rpm for an additional 2 h. Bacteria were washed and resuspended with sterile phosphate buffered saline (PBS) to a final concentration of 10^9^ colony-forming units (CFU)/ml. Bacteria were diluted to the desired concentrations for use in experiments.

### *In vivo* Experiments

Six weeks old mice were anesthetized by intraperitoneal injection of 10 mg/kg Rompun (Bayer, Seoul, Korea) and 50 mg/kg Zoletil (Virbac, Seoul, Korea). They were then intranasally (i.n.) inoculated with 30 μl of an *A. baumannii* (1 × 10^9^ CFU/ml) suspension in PBS. At the indicated times after infection, mice were anesthetized and blood was removed from cardiac puncture. Lungs and BAL fluids were collected 1 and 3 days post-infection to quantify immune cell populations, cytokines, and chemokines produced, and bacterial loads. For collection of BAL fluids, 1 mm of PBS was administered into lungs. After 3 to 5 times of lung massage, BAL fluids were collected without flushing. In a separate experiment using the same protocol, the right lobe of the lung was collected from each mouse and a lysate was prepared by homogenizing the tissue in PBS to determine bacterial growth. The left lung lobe was used to prepare slides for histopathological examination. Mouse recombinant IL-10 (750 ng/30 μl) was injected i.n. into mice 12 h after *A. baumannii* infection. Bone-marrow-derived macrophage (BMDMs) were isolated from WT and IL-10-deficient mice and cultured for 6 days. BMDMs (10^5^ cells/30 μl) obtained from WT mice or IL-10-deficient mice were transferred i.n. to WT or IL-10-deficient mice. At 24 h post-adoptive transfer, mice were challenged with *A. baumannii* (3 × 10^7^ CFU/30 μl) i.n. and euthanized 3 days after infection.

### Cell Culture and Bacterial Infection

BMDMs were prepared as previously described (15). Alveolar macrophages were also obtained from BAL fluids of 8–10 weeks old WT and IL-10-deficient mice as previously described (16). Thioglycollate-elicited neutrophils were isolated from the mouse peritoneal cavity as previously described (17). Briefly, mice were injected intraperitoneally with 2 ml of 4% thioglycollate broth (Sigma Aldrich, St. Louis, MO). Four hours later, 5 ml of sterile PBS was injected intraperitoneally and peritoneal lavage was performed. Red blood cells in the lavage were lysed with a buffer containing ammonium chloride and the total cell numbers were counted with a hemocytometer.

### Bacterial Counts in BAL Fluid and Lungs

Fifty microliters of serially-diluted BAL fluid or lung homogenate was spread onto LB agar plates supplemented with ampicillin (50 μg/ml). Following overnight culture at 37°C in an incubator, bacterial colonies were counted and the number of bacteria was expressed as CFU/ml of BAL fluids or CFU/g of lung tissue.

### Cell Counts in BAL Fluid

The total number of cells in the BAL fluid was counted using a hemocytometer. Differential cell counts based on morphologic criteria were performed using Diff-Quik staining.

### Histopathologic Examination

The left lung lobes were harvested and fixed in 10% neutral formalin for histopathological examination. The tissues were processed in an alcohol and xylene series and embedded in paraffin using routine procedures. Three-micrometer sections were prepared, stained with hematoxylin-eosin (H&E), and examined under a microscope. Histopathology of the lungs was blindly evaluated using an arbitrary scoring system depending on size of inflamed area composed of neutrophilic infiltration, loss of alveolar space, and necrotic lesions (score of 0 = no inflammation; 1 = inflamed area is 0–15% of sectioned lobes; 2 = 15–30%; 3 = 30–45%; 4 = 45–60%; 5 = 60–80%; and 6 = 80–100%).

### Lung Myeloperoxidase (MPO) Assay

Lung tissue was homogenized in 1 ml of myeloperoxidase buffer (50 mM potassium phosphate, pH 6.0). Homogenates were frozen and thawed twice. After centrifugation at 10,000 rpm for 10 min at 4°C, supernatants were collected and serially diluted. Diluted supernatant (100 μl) was added to 100 μl of assay buffer (50 mM potassium phosphate buffer containing 0.167 mg/ml O-dianisidine dihydrochloride and 0.0005% hydrogen peroxide). Absorbance values at a wavelength of 460 nm were measured between 1 and 10 min. Myeloperoxidase activity was calculated as the change in absorbance per min per milligram of total protein in the lysate.

### Measurement of Cytokines and Chemokines

The concentrations of IL-6, TNF-α, IL-1β, CXCL1, CXCL2, CCL2, and IL-10 in the lung homogenates and BAL fluids of *A. baumannii*-infected mice were determined using ELISA kits (R&D System, Minneapolis, MN, USA) according to the manufacturer's instructions.

### Phagocytic Activity and Bacterial Killing Ability of Macrophages

The phagocytosis of bacteria by macrophages was determined by the gentamicin protection assay (18). Briefly, BMDMs and neutrophils were seeded into 48-well plates at a density of 2 × 10^5^ cells/well and incubated at 37°C in an incubator with 5% CO_2_. Alveolar macrophages were seeded at a density of 2 × 10^4^ cells/well. The cells were pretreated without or with recombinant IL-10 (10 ng/ml) and subsequently infected with *A. baumannii* at an MOI of 1:10. After incubation for 60 min, cell membrane-impermeable antibiotic gentamicin (5 μg/ml) was added to the medium for 30 min to eliminate extracellular bacteria. At 1 (for phagocytosis) or 6 h (for bacterial killing) after infection, the cells were washed with PBS and subsequently lysed with 1% Triton X-100 in PBS. The cell lysate was plated onto LB agar supplemented with ampicillin (50 μg/ml) to determine the number of living bacteria engulfed by macrophages or neutrophils.

### Quantitative Real-Time PCR

Total RNA was extracted from BMDMs using easy-BLUE (Intron Biotechnology, Korea), according to the manufacturer's instructions. One microgram of total RNA was reverse transcribed to cDNA using ReverTra Ace qPCR RT Master Mix and cDNA Synthesis kit (Toyobo, Osaka, Japan). Equal amounts (1 μl) of cDNA were used for real-time PCR on a Rotor-Gene Q (Qiagen, Hilden, Germany) using a SYBR Green PCR kit (Qiagen). GAPDH was used for normalization. The following primers were used for real-time PCR: MARCO forward: 5′-ATCCTGCTCACGGCAGGTACT-3′; MARCO reverse: 5′-GCACATCTCTAGCATCTGGAGCT-3′; GAPDH forward: 5′-CAGTGGATGCAGGGATGATGTTCT-3′; GAPDH reverse: 5′-GTGGAGATTGTTGCCATCAACG-3′.

### Immunoblotting

For immunoblotting, BMDMs were seeded and incubated overnight in 6-well plates at a concentration of 2 × 10^6^ cells/well and infected with *A. baumannii* at an MOI of 10 by exposure for 24 h. Culture supernatants and remaining cells were mixed with a lysis buffer containing Nonidet P-40, complete protease inhibitor cocktail (Roche, Mannheim, Germany), and 2 mM dithiothreitol. Samples were separated by 8–12% sodium dodecyl sulfate-polyacrylamide gel electrophoresis (SDS-PAGE) and transferred to nitrocellulose membranes. The membranes were immunoblotted with primary antibodies, such as regular- and phospho-AKT, regular- and phosphor- STAT3 (each Cat No. #9272, #9271, #4904, and #9131; Cell Signaling Technology), and anti-β-actin (Cat No. sc-47779; Santa Cruz Biotechnology, Inc., Santa Cruz, CA, USA). After immunoblotting with secondary antibodies (goat anti-rabbit IgG-HRP, Cat No. sc-2357; Santa Cruz Biotechnology), proteins were visualized via an enhanced chemiluminescence (ECL) reagent from BioRad (Hercules, CA, USA).

### Statistical Analysis

Differences between groups were determined by two-tailed Student's *t*-tests or one-way analysis of variance, followed by *post-hoc* analysis (Newman–Keuls multiple comparison test). All statistical analyses were performed using GraphPad Prism 5 (GraphPad Software Inc., La Jolla, CA, USA). Statistical significance was considered at *P* < 0.05.

## Results

### IL-10 Protects Mice From Lethality by Lung Infection With *A. baumannii* and Promotes Bacterial Clearance

**Figure 1:**
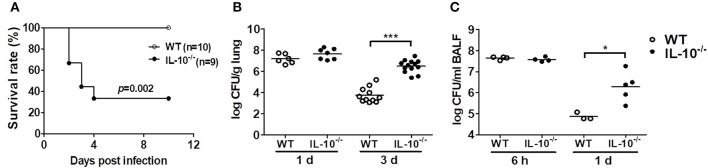
Deficiency of IL-10 increases mortality and pulmonary bacterial loads in mice after *A. baumannii* infection. WT and IL-10-deficient mice were infected i.n. with *A. baumannii* (3 × 10^7^ CFU/30 μl) and euthanized at 6 h, 1 day, or 3 days after infection. **(A)** Survival was monitored daily. **(B,C)** Bacterial loads in lung homogenates and BALF were counted by plating assays. *P < 0.05 and ***P < 0.001.

To determine the role of IL-10 in host defenses against *A. baumannii* infection, WT and IL-10-deficient mice were infected i.n. with the bacteria, and the survival rates and bacterial burden in the lungs and BAL fluids were evaluated. Infection with 1.5 × 10^7^ CFU of the bacteria did not induce lethality of any mice (data not shown). When infected with 3 × 10^7^ CFU, only 33% of the IL-10-deficient mice survived, whereas no WT mice died until the end of the experiment (Figure 1A). Bacterial loads in the lung homogenates were comparable between WT and IL-10-deficient mice 1 day after infection. However, the bacterial loads in IL-10-deficient mice were significantly higher than those in WT mice at day 3 (Figure 1B). In addition, the bacterial load in BAL fluids was significantly higher in IL-10-deficient mice compared to those in the WT mice 1 day after infection, although there was no significant difference between two groups at 6 h (Figure 1C). On day 3, bacteria were not detectable in BAL fluids from any mice (data not shown).

### IL-10 Reduces the Lung Pathology and Production of Proinflammatory Cytokines in *A. baumannii*-Infected Mice

**Figure 2:**
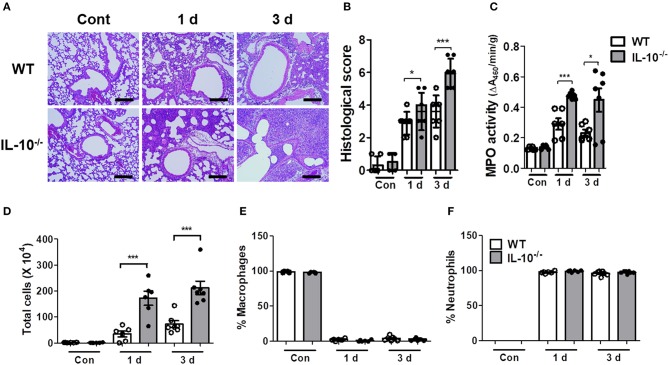
IL-10-deficient mice show severe lung pathology after *A. baumannii* infection. WT and IL-10-deficient mice were infected i.n. with *A. baumannii* (3 × 10^7^ CFU/30 μl) and euthanized at 1 day and 3 days after the infection. **(A,B)** Lung histopathology was evaluated in H&E-stained sections and scored as described in the Materials and Methods. Bar = 100 μm **(C)** MPO activities in lung homogenates of WT and IL-10-deficient mice were measured. **(D)** The total number of BAL fluid cells was determined with a hemocytometer. **(E,F)** Differential counts of macrophages and neutrophils were obtained by Diff-Quik staining. **(B–F)** Results are combined from two independent experiments and presented as means ± SD. *P < 0.05 and ***P < 0.001.

As excessive production of cytokines can lead to tissue damage and severe pathology, histopathological examination was performed on the lungs of mice infected with *A. baumannii*. IL-10-deficient mice showed more severe lung pathology and higher histology scores compared to WT mice at days 1 and 3 (Figures 2A,B). The activity of myeloperoxidase (MPO), mostly expressed in neutrophils, was also significantly increased in the lung homogenates of IL-10-deficient mice with *A. baumannii* infection at 1 and 3 days after infection (Figure 2C). Furthermore, total cell counts in BAL fluids were increased in IL-10-deficient mice, with no difference in the composition ratio of macrophages and neutrophils (Figures 2D–F). Most inflammatory cells in BAL fluids were neutrophils, which was confirmed by flow cytometric analysis (Figure 2F and Supplementary Figures 1A,B).

The levels of proinflammatory cytokines or chemokines, including IL-6, TNF-α, IL-1β, CXCL1, CXCL2, and CCL2 in the lung homogenates and BAL fluids, were significantly higher in IL-10-deficient mice, depending on the experimental time points, compared to WT mice (Table 1). The levels of IL-6, TNF-α, and IL-1β in BAL fluids and lung homogenates of both uninfected WT and IL-10-deficient mice were undetectable range in our system (<15.625 pg/ml) (data not shown). CXCL1, CXCL2, and CCL2 levels were also < 100 pg/ml in both groups and there was no significant difference between two groups (data not shown).

**Table 1:** Deficiency of IL-10 increases cytokines and chemokines production in response to *A. baumannii* infection.

	**Lung homogenates**				**BAL fluids**			
	**1 day**		**3 days**		**6 h**		**1 day**	
**ng/ml**	**WT**	**IL-10^**−/−**^**	**WT**	**IL-10^**−/−**^**	**WT**	**IL-10^**−/−**^**	**WT**	**IL-10^**−/−**^**
IL-6	5.2 ± 0.8	15.9 ± 1.8^***^	1.1 ± 0.7	8.9 ± 4.2^*^	3.1 ± 0.8	2.3 ± 1.7	1.3 ± 0.8	3.2 ± 0.4^*^
TNF-α	2.2 ± 0.9	6.5 ± 1.6^***^	0.3 ± 0.1	3.2 ± 1.1^*^	12.3 ± 1.0	10.6 ± 1.1	2.3 ± 0.8	7.6 ± 3.4^*^
IL-1β	11.2 ± 3.6	17.1 ± 2.4^*^	7.8 ± 2.9	9.5 ± 2.8	0.9 ± 0.4	0.7 ± 0.3	1.0 ± 0.1	1.1 ± 0.6
CXCL1	20.5 ± 3.9	41.6 ± 2.6^***^	5.7 ± 1.0	22.1 ± 7.8^*^	1.3 ± 0.01	1.3 ± 0.02	0.8 ± 0.1	1.2 ± 0.1^**^
CXCL2	14.1 ± 1.9	28.6 ± 13.5	30.2 ± 16.8	34.7 ± 10.1	0.3 ± 0.1	12.3 ± 1.8^***^	1.4 ± 0.7	8.0 ± 3.8^*^
CCL2	4.2 ± 0.7	5.1 ± 0.6	3.6 ± 0.7	5.1 ± 0.4^*^	0.1 ± 0.03	0.07 ± 0.02^*^	0.2 ± 0.03	0.1 ± 0.02^***^

* *P < 0.05*,

** P < 0.01, and

*** *P < 0.001*.

### Intranasal Administration of Recombinant IL-10 Rescues IL-10-Deficient Mice Against *A. baumannii* Infection

**Figure 3:**
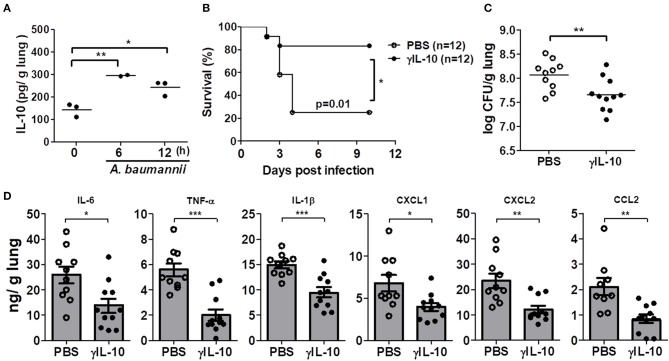
Recombinant IL-10 rescues mortality, bacterial clearance and anti-inflammation in *IL-10*-deficient mice after infection with *A. baumannii*. IL-10-deficient mice were administered with γIL-10 (750 ng/30 μl) i.n. 12 h after *A. baumannii* infection (3 × 10^7^ CFU/30 μl) and lungs were collected 1 day after infection. **(A)** The production of IL-10 in *A. baumannii*-infected WT mice in a time-dependent manner. **(B)** Survival was monitored for 10 days. **(C)** Bacterial loads in lung homogenates were counted by plating assays. **(D)** Levels of IL-6, TNF-α, IL-1β, CXCL1, CXCL2, and CCL2 were measured by ELISA. **(C,D)** Results are combined from three independent experiments and presented as means ± SD. *P < 0.05, **P < 0.01, and ***P < 0.001.

We further investigated whether administration of γIL-10 could protect IL-10-deficient mice from the lethality of an *A. baumannii* infection and improve bacterial clearance in the mice lungs. First, the time-dependent response of *in vivo* IL-10 production in the lungs of mice in response to *A. baumannii* infection was determined. IL-10 was significantly induced 6 h after the bacterial infection and its level was sustained up to 12 h after the infection (Figure 3A). Therefore, γIL-10 was administered i.n. to IL-10-deficient mice 12 h after *A. baumannii* infection, and mice survival and bacterial CFUs in the lungs were determined. γIL-10 treatment enhanced the survival of the IL-10-deficient mice infected with *A. baumannii* and reduced the bacterial burdens in the mice lungs at day 1 (Figures 3B,C). In addition, levels of IL-6, TNF-α, IL-1β, CXCL1, CXCL2, and CCL2 in the lung homogenates were significantly lower in mice treated with γIL-10 (Figure 3D). Taken together, IL-10 seemed to have protective host effect against pulmonary infection with *A. baumannii* by suppressing the lung inflammation and enhancing bacterial clearance.

### IL-10 Promotes Phagocytosis and Bacterial Killing Ability of Macrophages in Response to *A. baumannii* by Regulating the Expression of a Scavenger Receptor MARCO

**Figure 4:**
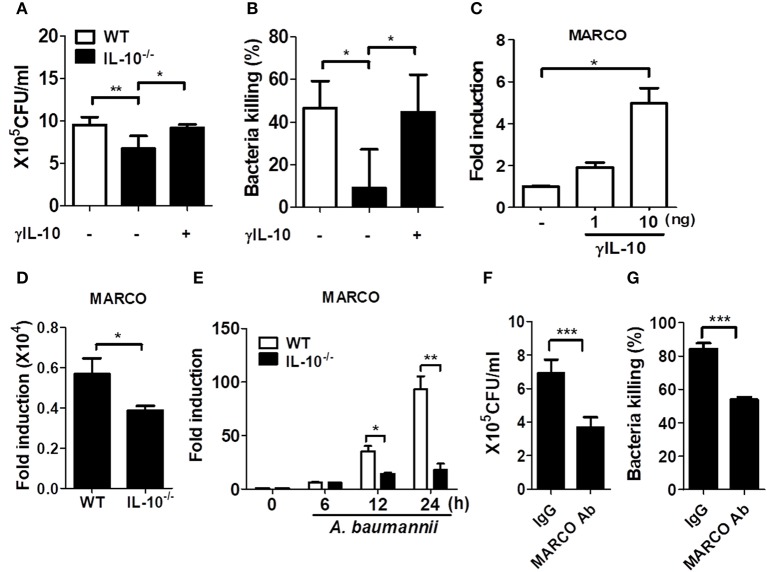
IL-10 deficiency leads to impaired phagocytosis and bacterial killing of macrophages in response to *A. baumannii*. **(A,B)** BMDMs from WT and IL-10-deficient mice were infected with *A. baumannii* at 1/10 MOI followed by gentamicin treatment 60 min after infection to remove extracellular bacteria. Live bacteria were then counted by plating onto LB agar supplemented with ampicillin (50 μg/ml) at 1 h (for phagocytosis) or 6 h (for bacterial killing) after infection with *A. baumannii*. **(A–C)** BMDMs were pretreated with 10 ng/ml of γIL-10 for 2 h and subsequently infected with *A. baumannii*. **(C)** Expression levels of MARCO mRNA in γIL-10 treated BMDMs at 24 h. **(D)** Expression levels of endogenous MARCO mRNA in WT and IL-10-deficient BMDMs at 24 h. **(E)** Expression levels of MARCO mRNA in *A. baumannii*-infected BMDMs at indicated time points were evaluated by real-time PCR. Fold increase (arbitrary unit) compared to levels in uninfected BMDMs are presented. Results are from one representative experiment of three independent experiments. **(F,G)** For neutralizing antibody assays, BMDMs were pretreated with 0.2 ug/ml of goat IgG control or MARCO mAb for 2 h, then infected with *A. baumannii* and analyzed for phagocytic and bacterial killing abilities by gentamicin protection assays. *P < 0.05, **P < 0.01, and ***P < 0.001.

The *in vivo* results imply that the ability to clear *A. baumannii* is likely impaired in IL-10-deficient immune cells, such as macrophages and neutrophils, as the bacterial load in the lungs was increased in IL-10-deficient mice (Figures 1B,C), although the number of recruited immune cells was higher in those mice (Figure 2D). We thus compared the phagocytic and bacterial killing abilities of macrophages and neutrophils from WT and IL-10-deficient mice against *A. baumannii*. The bacterial phagocytic and killing abilities were impaired in IL-10-deficient BMDMs and were restored by pretreatment with γIL-10 (Figures 4A,B). Consistently, the ability to phagocytose *A. baumannii* was also impaired in IL-10-deficient alveolar macrophages, which was restored by γIL-10 (Supplementary Figure 2). In this experiment, bacterial killing ability could not be assessed due to use of low number of cells, resulting that no colony was detectable in both WT and IL-10-deficient cells at 6 h. In neutrophils, an IL-10 deficiency led to impairment of phagocytosis against *A. baumannii*, but there was no significant difference in the bacterial killing ability between WT and IL-10-deficient neutrophils (Supplementary Figures 3A,B). Therefore, we focused on determining the function of macrophages in controlling *A. baumannii* infections in further experiments.

Previous studies showed that macrophages exposed to IL-10 exhibited increased expression of macrophage receptor with collagenous structure (MARCO) (19, 20), which is responsible for phagocytosis and *in vivo* clearance of pathogenic bacteria (21–23). In fact, γIL-10 treatment increased the gene expression of MARCO in BMDMs at 24 h (Figure 4C). We further examined MARCO expression in response to *A. baumannii* infection in WT and IL-10-deficient BMDMs. Remarkably, endogenous MARCO expression was slightly reduced in IL-10-deficient BMDMs, compared to WT cells, although its expression level was very weak in both intact cells (Figure 4D). *A. baumannii* infection increased the gene expression of MARCO time-dependently in both WT and IL-10-deficient cells, but the levels were significantly lower in IL-10-deficient cells (Figure 4E). We next examined the effect of MARCO on the phagocytic and bacterial killing abilities of macrophages in response to *A. baumannii* using an anti-MARCO antibody. Results showed that pretreatment with the anti-MARCO antibody reduced the phagocytic and bacterial killing abilities of *A. baumannii*-infected macrophages (Figures 4F,G). These findings indicate that IL-10 may play an important role in the removal of *A. baumannii* from the host by promoting phagocytosis and bacterial killing by macrophages through a MARCO-dependent pathway.

### IL-10 Deficiency Leads to Impaired Phosphorylation of STAT3 in Macrophages in Response to *A. baumannii*, Which Contributes to Phagocytosis and Bacterial Killing by Regulating MARCO Expression

**Figure 5:**
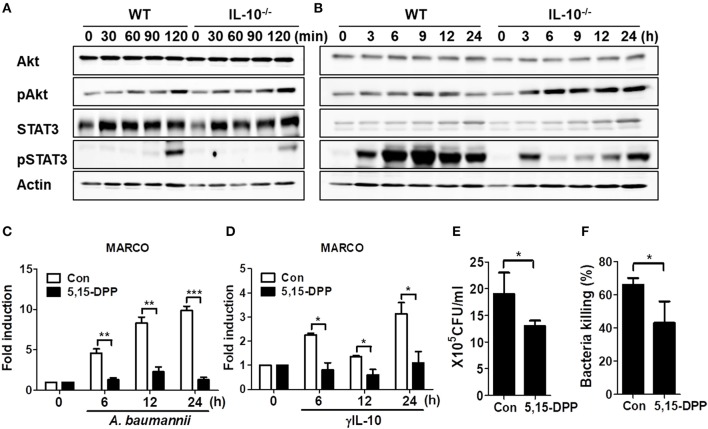
IL-10 plays a significant role in *A. baumannii* induced MARCO expression via the activation of STAT3. **(A,B)** WT and IL-10-deficient BMDMS were infected with *A. baumannii* at 1/10 MOI for the indicated times. Cells were harvested and analyzed for AKT and STAT3 phosphorylation by Western blotting. Results are from one representative experiment of two independent experiments. **(C,D)** Inhibition of STAT3 inhibition by pretreatment with D,15-DPP (100 uM) 2 h prior to treatment with *A. baumannii* (MOI 1/10) and γIL-10 (10 ng/ml). Expression levels of MARCO mRNA in *A. baumannii*-infected BMDMs were evaluated by real-time PCR. **(E,F)** BMDMs were pretreated with D,15-DPP (100 uM) for 2 h, then infected with *A. baumannii* and analyzed for phagocytic and bacterial killing abilities by gentamicin protection assays. *P < 0.05, **P < 0.01, and ***P < 0.001.

IL-10 signaling predominantly occurs via the activation of AKT or STAT3-mediated signaling pathways (24, 25). Thus, we investigated phosphorylation of AKT and STAT3 in WT and IL-10-deficient mice BMDMs infected with *A. baumannii*. Results showed that *A. baumannii*-induced AKT phosphorylation was unimpaired in IL-10-deficient macrophages, compared to WT cells (Figures 5A,B and Supplementary Figures 4A,C), but showed slightly enhanced activation in IL-10-deficient cells after long infection times (Figure 5B and Supplementary Figure 4C). Remarkably, *A. baumannii* strongly induced STAT3 phosphorylation in WT macrophages after 2 h of infection but was reduced in IL-10-deficient cells (Figures 5A,B and Supplementary Figures 4B,D). Next, we determined whether STAT3 activation was responsible for *A. baumannii*-induced MARCO expression in macrophages and their phagocytic and bacterial killing abilities. As shown in Figure 5C, the gene expression of MARCO induced by *A. baumannii* was abolished by treatment with a specific Stat3 inhibitor, D,15-DPP. D,15-DPP also inhibited γIL-10-induced expression of MARCO in macrophages (Figure 5D). Phagocytosis and bacterial killing by macrophages infected with *A. baumannii* were also reduced by D,15-DPP treatment (Figures 5E,F). Therefore, it is likely that IL-10 upregulated MARCO expression by activating STAT3-mediated signaling, which mediated bacterial clearance in macrophages infected with *A. baumannii*.

### Intranasal Adoptive Transfer of WT Macrophages Improves Survival of IL-10-Deficient Mice Infected With *A. baumannii*

**Figure 6:**
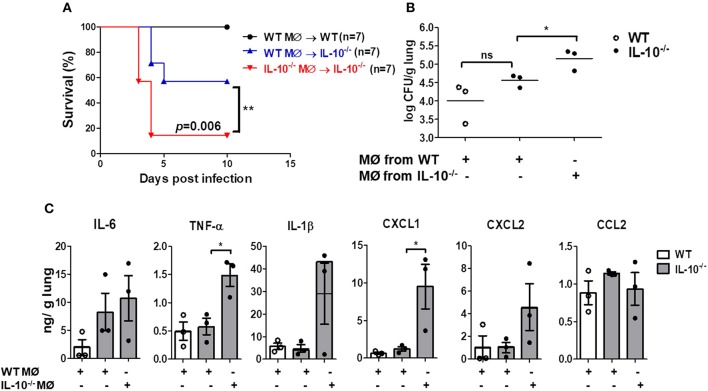
Reduced mortality, bacterial loads and cytokines production in lungs of IL-10-deficient mice infected with *A. baumannii* after adoptive transfer of WT BMDMs. 1 × 10^5^ cells/30 μl BMDMs from WT or IL-10-deficient mice were transferred i.n. into either WT or IL-10-deficient mice (donor→ recipient). Mice were allowed to rest for 24 h, then were infected with *A. baumanii* (3 × 10^7^ CFU/30 μl) i.n. and euthanized 3 days after infection. **(A)** Survival was monitored for 10 days. **(B)** Bacterial loads in lung homogenates were counted by plating assays. **(C)** Levels of IL-6, TNF-α, IL-1β, CXCL1, CXCL2, and CCL2 were measured by ELISA. Results are expressed as means ± SD. *P < 0.05.

We finally sought to determine whether local adoptive transfer with WT macrophages could rescue the IL-10-deficient mice from pulmonary infection with *A. baumannii*. WT or IL-10-deficient BMDMs (1 × 10^5^ cells/animal) were adoptively transferred into WT and IL-10-deficient recipient mice through an intranasal route 24 h before infection. IL-10-deficient mice receiving BMDMs from WT mice showed significantly increased survival and bacterial clearance compared to mice who received BMDMs from IL-10-deficient mice (Figures 6A,B). In addition, *A. baumannii*-induced production of TNF-α and CXCL1 in the lung homogenates was significantly lower in IL-10-deficient mice who received WT BMDMs compared to the mice who received IL-10-deficient cells, although there was no significant difference in IL-6, IL-1β, CXCL2, and CCL2 levels between the two groups (Figure 6C).

## Discussion

IL-10 exerts both harmful and beneficial effects in host defenses against bacterial infections, depending on their strains. IL-10 negatively affects mice infected with *P. aeruginosa, B. parapertussis*, and *K. pneumonia* due to decreased bacterial clearance, impaired recruitment of innate immune cells, and reduced production of proinflammatory cytokines/chemokines (7–9). In contrast, IL-10 protects hosts from pneumonia by *C. kutscheri* and *S. pneumoniae* infections by alleviating excessive lung inflammation (10, 11). In the present study of mice with pulmonary infections of *A. baumannii*, we showed that IL-10 deficiency resulted in enhanced mortality, severe lung inflammation, and excess production of proinflammatory cytokines and chemokines in lung homogenates. Intranasal administration of γIL-10 or adoptive transfer of macrophages capable of producing IL-10 improved mice survival and promoted bacterial clearance in IL-10-deficient mice. Consistent with a study by Noto et al. (14), our results clearly indicated that IL-10 has beneficial effects on host defenses against pulmonary infection with *A. baumannii*.

Neutrophils and macrophages are known to play important roles in host resistance to pulmonary infection with *A. baumannii* (26, 27). The number of both cells in the BAL fluids of mice was increased 24 h after pulmonary infection with *A. baumannii*, although the inflammatory cells in the BAL fluids were largely composed of neutrophils (26). A previous study reported that neutrophil depletion using monoclonal antibodies led to decreased mice survival and impaired bacterial clearance in the lungs (27). Moreover, *in vivo* depletion of alveolar macrophages using clodronate liposomes increased bacterial burdens in the lungs and BAL fluids of mice with intranasal challenges of *A. baumannii* and reduced production of proinflammatory cytokines and chemokines (26). In the present study, the total number of cells in BAL fluids were significantly higher in IL-10-deficient mice infected with *A. baumannii*, compared to WT mice, with a large proportion of neutrophils. Nevertheless, the bacterial burdens in the lung homogenates and BAL fluids were higher in IL-10-deficient mice, suggesting that the ability of immune cells to control the growth of *A. baumannii* may be impaired in IL-10-deficient mice. In fact, our *in vitro* experiments showed that an IL-10 deficiency reduced the phagocytosis and bacterial killing of macrophages against *A. baumannii*, which was restored by γIL-10 treatment. Moreover, the neutrophils required IL-10 for optimal phagocytosis, although IL-10 did not affect the bacterial killing ability of the neutrophils. A previous study showed that IL-10 suppressed PGE_2_ production, which resulted in enhanced clearance of *E. coli* by promoting the bacterial phagocytosis by neutrophils and macrophages through a CR3-dependent manner in a meningitis model (13). Therefore, it is likely that neutrophils, as well as macrophages, play important roles in IL-10-mediated host defenses against *A. baumannii*, although we did not check the *in vivo* role of neutrophils in this study. Further study is needed to determine the *in vivo* role of neutrophils in IL-10-mediated host defenses against *A. baumannii* infections.

Macrophages contribute to host defenses against microbial infections through phagocytosis. MARCO is a scavenger receptor on the surface of macrophages that mediates phagocytosis of unopsonized particles, bacteria, and oxidized lipids (16, 28, 29). Transcriptome analysis by next-generation sequencing revealed that IL-10 stimulation enhanced expression of scavenger receptors, including MARCO, in macrophages (20). IL-10-polarized M2 macrophages displayed enhanced phagocytic activity against *H. ducreyi* and similar bacterial killing (30). In this study, we showed that MARCO expression in response to *A. baumannii* decreased in IL-10-deficient macrophages, and blocking it using antibodies impaired phagocytosis and bacterial killing in WT macrophages, suggesting that IL-10 may participate in host protection against *A. baumannii* infection by regulating such macrophage functions as phagocytosis and bacterial killing through a MARCO-dependent pathway. In fact, intranasal adoptive transfer of WT macrophages into IL-10-deficient mice enhanced mice survival and reduced bacterial burdens in the lungs of mice infected with *A. baumannii*, indicating that macrophages may be a critical immune cell regulating the IL-10-mediated host defense against *A. baumannii* infections.

It is generally accepted that IL-10 exerts its biological effects by interacting with a specific cell surface receptor, IL-10R (31, 32). The binding of IL-10 to its receptor leads to the activation of diverse signaling pathways, including JAK/STAT and PI3K/AKT, with different cellular functions (31–34). How IL-10 regulates MARCO expression in macrophages is not yet known. A previous study showed that LPS was capable of inducing a delayed activation of STAT3 that was dependent on the production of endogenous IL-10 (34). In this study, *A. baumannii* induced STAT3 phosphorylation in WT BMDMs starting 2 h after infection, a process which was impaired in IL-10-deficient macrophages. In addition, treatment with a STAT3 specific inhibitor, 5,15-DPP, reduced *A. baumannii*-induced expression of MARCO and impaired phagocytosis and bacterial killing in macrophages. Therefore, IL-10 mediated bacterial clearance by promoting the phagocytosis and bacterial killing of macrophages through STAT3-dependent MARCO expression. In addition, Wu et al. recently showed that AKT activation led to increased MARCO expression in macrophages, which improved phagocytosis in IFN-γ-treated cells (35). However, in this study, IL-10 deficiency did not influence AKT phosphorylation in response to *A. baumannii*. Accordingly, AKT-mediated signaling is not likely to a play major role in the IL-10-mediated host defenses against *A. baumannii*.

In conclusion, we showed that IL-10 exerted protective effects against pulmonary infection with *A. baumannii* by promoting mice survival and bacterial clearance, and by reducing the lung pathology. IL-10 may improve mice survival by suppressing excessive inflammation rather than improving bacterial clearance by macrophages, as difference of bacterial clearance in the lungs were seen at late time of infection (3 days) and supra physiological levels of IL-10 was needed to improve ability of bacterial phagocytosis and killing of immune cells. Nevertheless, our study clearly show that IL-10 mediates phagocytosis and bacterial killing of macrophages in response to *A. baumannii* by upregulating MARCO expression via STAT3-mediated signaling. The IL-10-mediated regulation of macrophage function is likely limited to some bacteria, including *A. baumannii*, as the bacterial burdens were reduced in the lungs of IL-10-deficient mice infected with *S. pneumoniae*, but the lung inflammation was still more severe in the *S. pneumoniae*-infected mice (11). In addition, in a study by de Breij et al., pathogenic strains of *A. baumannii* led to less production of IL-10 in the lungs of infected mice (36), suggesting that *A. baumannii* likely exerts its pathogenicity by regulating IL-10 production in host cells. The microbial factors and relevant mechanisms regulating host IL-10 production in response to *A. baumannii* infection also remain to be determined.

## Ethics Statement

Protocols for animal studies were approved by the Institutional Animal Care and Use Committee of Chonnam National University (Gwangju, Korea) (Approval number: CNU IACUC-YB-2015-33).

## Author Contributions

M-JK acquired, analyzed, and interpreted data as well as developed study concept and wrote manuscript. A-RJ, J-YP, J-HA, T-SL, D-YK, M-SL, SH, and Y-JJ wrote sections of the manuscript. J-HP developed the study concept, obtained funding and ethics, interpreted data, and wrote manuscript. All authors read and approved the final manuscript.

### Conflict of Interest

The authors declare that the research was conducted in the absence of any commercial or financial relationships that could be construed as a potential conflict of interest.
