# Supplementary material for: IL-10 Protects Mice From the Lung Infection of Acinetobacter baumannii and Contributes to Bacterial Clearance by Regulating STAT3-Mediated MARCO Expression in Macrophages
Source: Front Immunol. 2020 Feb 21;11:270. doi: 10.3389/fimmu.2020.00270 (PMC7047127; doi:10.3389/fimmu.2020.00270)
Supplement: Supplementary file 1 [file Image_1.pdf]

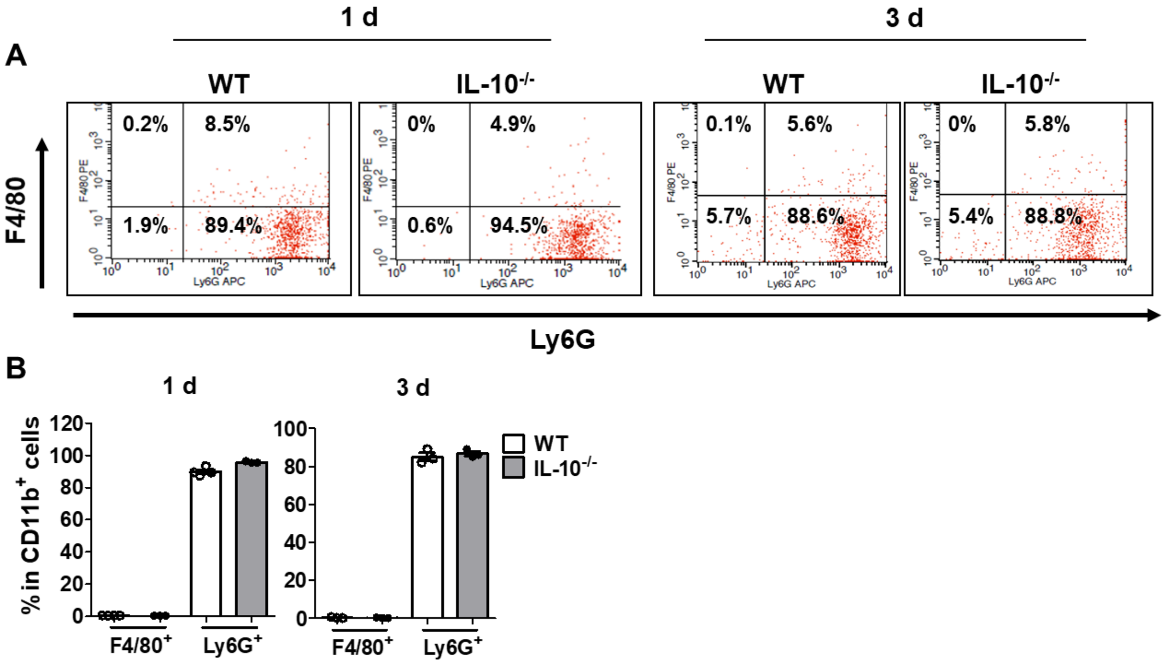

Supplementary Figure 1. Flow cytometric analysis to determine cell populations in BAL fluids. WT and IL-10-deficient mice (n=3 or 4) were infected with *A. baumannii* and BAL fluids were collected at indicated times after infection. They were washed in ice-cold flow cytometry buffer (2% bovine serum albumin and 2 mM EDTA in PBS, pH 7.5), incubated with each antibody for 15 min, and washed twice with flow cytometry buffer. The cells were stained with mAbs including CD11b-PE-cy7 (clone M1/70, Cat No. 552850; BD Biosciences; Pharmingen, San Diego, USA) and Ly6G-APC (clone 1A8, Cat No. 560599; BD Biosciences) and F4/80-FITC (clone bm8, Cat No. 11-4801-81; eBioscience, San Diego, CA, USA). (A) F4/80$^+$ and Ly6G$^+$ populations were systematically gated from CD11b+ population. (B) A population of F4/80$^+$ and Ly6G$^-$ was defined as macrophages and F4/80$^-$ and Ly6G$^+$ cells were as neutrophils.
