# Supplementary material for: IL-10 Protects Mice From the Lung Infection of Acinetobacter baumannii and Contributes to Bacterial Clearance by Regulating STAT3-Mediated MARCO Expression in Macrophages
Source: Front Immunol. 2020 Feb 21;11:270. doi: 10.3389/fimmu.2020.00270 (PMC7047127; doi:10.3389/fimmu.2020.00270)
Supplement: Supplementary file 2 [file Image_2.pdf]

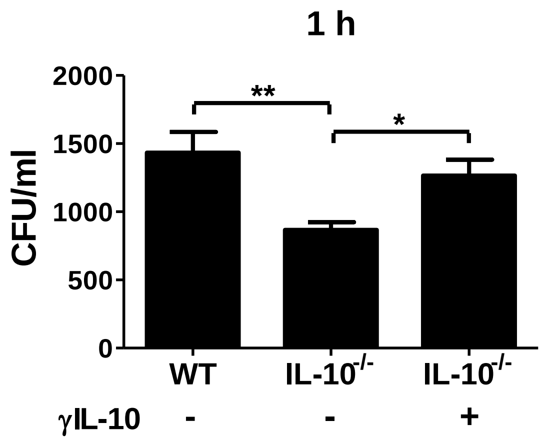

Supplementary Figure 2. IL-10 enhances the bacterial phagocytosis ability of alveolar macrophages infected with *A. baumannii*. (A, B) Alveolar macrophages from WT and IL-10-deficient mice were seeded at a density of 2×10^4 cells/well and infected with *A. baumannii* at 1/10 MOI followed by gentamicin treatment 60 min after infection to remove extracellular bacteria. Live bacteria were then counted by plating onto LB agar supplemented with ampicillin (50 μg/ml) at 1 h after infection. Results are expressed as means ± SD. **P*<0.05, ***P*<0.01.
