# Supplementary material for: IL-10 Protects Mice From the Lung Infection of Acinetobacter baumannii and Contributes to Bacterial Clearance by Regulating STAT3-Mediated MARCO Expression in Macrophages
Source: Front Immunol. 2020 Feb 21;11:270. doi: 10.3389/fimmu.2020.00270 (PMC7047127; doi:10.3389/fimmu.2020.00270)
Supplement: Supplementary file 3 [file Image_3.pdf]

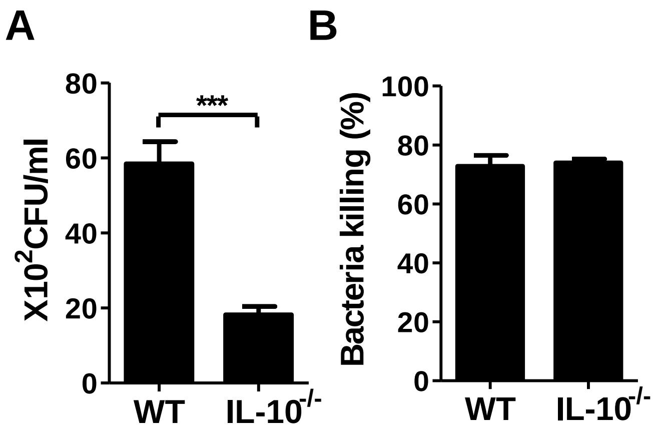

Supplementary Figure 3. IL-10 deficiency leads to impaired phagocytosis of neutrophils in response to *A. baumannii*. (A, B) Thioglycollate-elicited neutrophils (Nphs) from WT and IL-10-deficient mice were infected with *A. baumannii* at 1/10 MOI followed by gentamicin treatment 60 min after infection to remove extracellular bacteria. Live bacteria were then counted by plating onto LB agar supplemented with ampicillin (50 μg/ml) at 1 h (for phagocytosis) or 6 h (for bacterial killing) after infection with *A. baumannii*. Results are expressed as means ± SD. ***P<0.001.
