# Supplementary material for: IL-10 Protects Mice From the Lung Infection of Acinetobacter baumannii and Contributes to Bacterial Clearance by Regulating STAT3-Mediated MARCO Expression in Macrophages
Source: Front Immunol. 2020 Feb 21;11:270. doi: 10.3389/fimmu.2020.00270 (PMC7047127; doi:10.3389/fimmu.2020.00270)
Supplement: Supplementary file 4 [file Image_4.pdf]

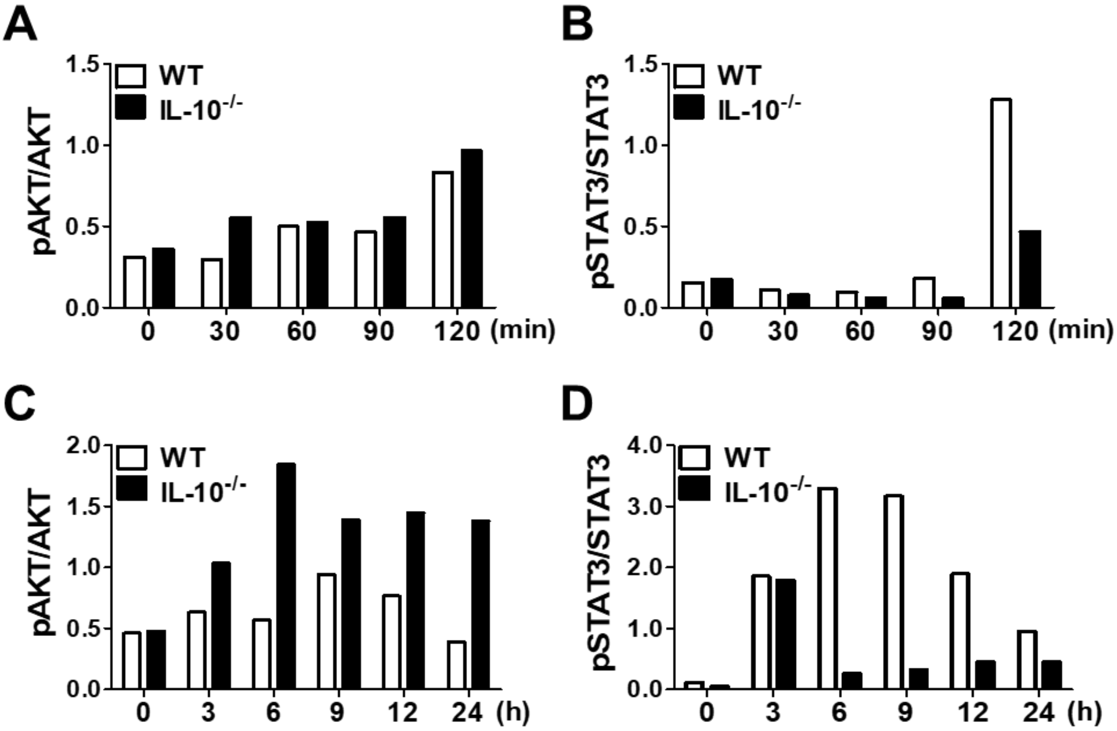

Supplementary Figure 4. ***A. baumannii*-induced phosphorylation of AKT and STAT3 in WT and IL-10-deficient macrophages.** Immunoblotting was performed as described in Materials and Methods. (A-D) Density of each band in Figures 5A, B was measured by Image J software and the ratio of pATK/AKT and pSTAT3/STAT3 was presented.
